# The complete mitochondrial genome of Daurian redstart *Phoenicurus auroreus* (Aves: Passeriformes: Muscicapidae)

**DOI:** 10.1080/23802359.2020.1770139

**Published:** 2020-05-28

**Authors:** Chao Du, Li Liu, Yunpeng Liu, Zhaohui Fu, Yongtao Xu

**Affiliations:** a Baotou Teachers’ College, Inner Mongolia University of Science & Technology, Baotou, Inner Mongolia, People’s Republic of China; b College of Forestry, Jiangxi Agricultural University, Nanchang, Jiangxi, People’s Republic of China

**Keywords:** *Phoenicurus auroreus*, mitochondrial genome, phylogeny, Muscicapidae

## Abstract

We sequenced the mitochondrial genome of Daurian redstart *Phoenicurus auroreus* using the next-generation sequencing. The circular genome is 16,832 bp long, encoding 13 protein-coding genes (PCGs), 22 transfer RNAs (tRNAs), 2 ribosomal RNAs (rRNAs), and a control region. The lengths of *rrnL* and *rrnS* were determined to be 1,597 and 984 bp, respectively. The phylogenetic analysis of 18 mitogenomes of Muscicapidae supports monophylies of all genera, including *P. auroreus*. The information would be useful for understanding the phylogeny and evolution of Muscicapidae.

The Daurian redstart (*Phoenicurus auroreus*) is a small passerine bird belongs to the family Muscicapidae, and is widely distributed in a large range of temperate Asia (BirdLife International 2020). The taxonomic status of *P. auroreus*, as well as some other lineages of the family, remain controversial (Tu et al. 2019; Chen et al. 2020). Here, we sequenced and assembled the complete mitochondrial genome of *P. auroreus*. The information would be helpful to infer the phylogenetic relationships of Muscicapidae.

The sample of *P. auroreus*, which was died of natural causes, were collected at Guyang, Baotou, Inner Mongolia, China (40°53′N, 110°13′E). The specimens were stored in the Museum of Baotou Teachers’ college (BA850127, Baotou Teachers’ college, China). Total genomic DNA was extracted using the DNeasy tissue kit (QIAGEN) following the manufacturer’s standard protocol. The library was constructed using an Illumina TruSeq Library Preparation kit and sequenced on an Illumina HiSeq 2500 platform. The raw data were trimmed using fastp (Chen et al. 2018) to remove adapters and low-quality reads. High-quality reads were assembled using MITObim v1.9.1 (Hahn et al. 2013), and annotated using MitoZ (Meng et al. 2019). The annotated mitogenome has been deposited in GenBank database under the accession number of MT366880.

The complete mitochondrial genome of *P. auroreus* is a typical circular DNA molecule with 16,832 bp in length, encoding 13 protein-coding genes (PCGs), 22 transfer RNAs (tRNAs), 2 ribosomal RNAs (rRNAs), and a control region. the A–T content of *P. auroreus* mitogenome is 56.30%, which is the moderate of the known flycatcher mitogenomes. The major strand (J strand) carried most of the genes (12 PCGs and 14 tRNAs), while only nad6 had the remaining eight tRNAs which were on the minor strand (N strand). Most PCGs initiated with typical ATG start codons, except for *cox1* initiating with GTG. The lengths of *rrnL* and *rrnS* were determined to be 1597 and 984 bp, respectively.

Eighteen complete mitogenomes of Muscicapidae, including *P. auroreus*, were used to infer the phylogenetic relationships among flycatchers based on the maximum likelihood (ML) analysis utilizing IQ-TREE 2 (Minh et al. 2019). The resulting tree supports monophylies of all genera, including *P. auroreus* in the present study (Figure 1). The sequence data would be useful for elaboration of the phylogeny and evolution of Muscicapidae.

**Figure 1.**
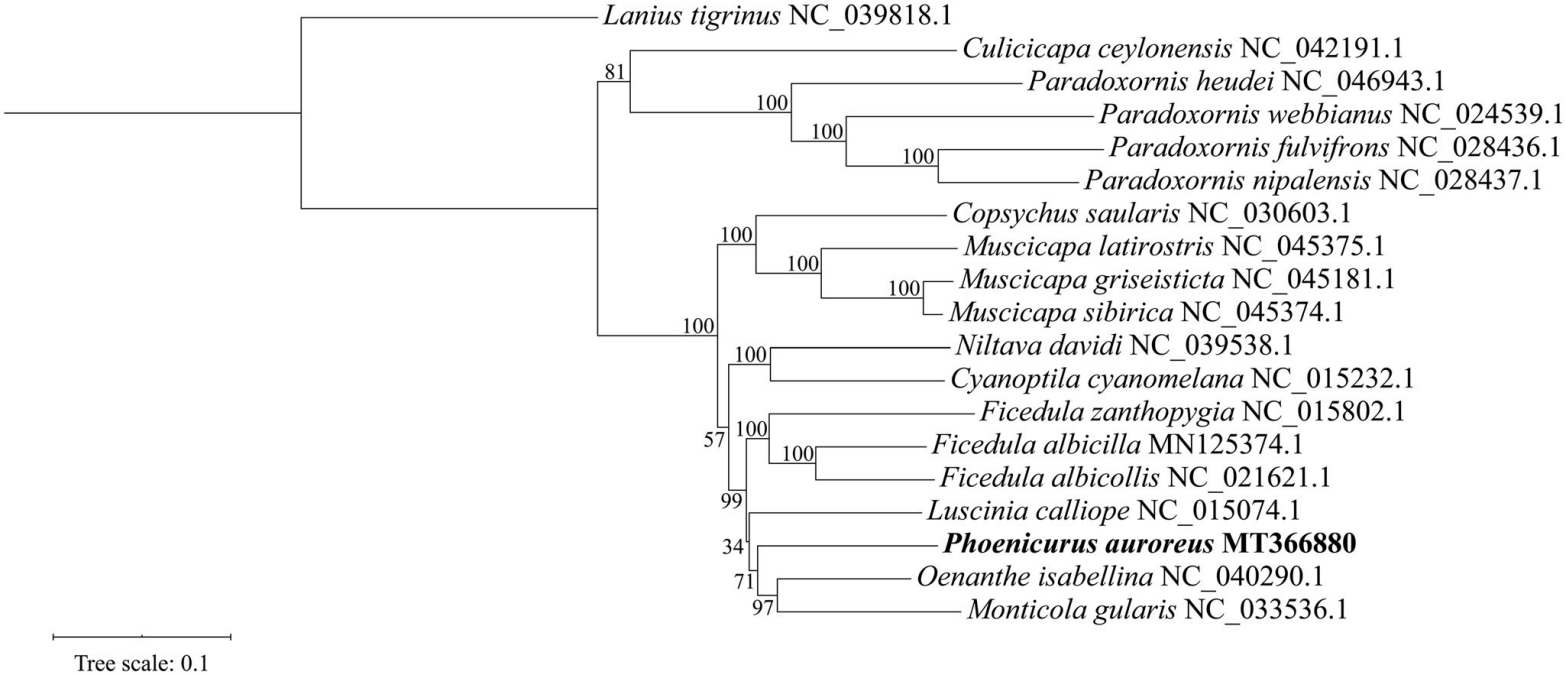
The maximum likelihood tree of 18 Muscicapidae taxa based on the nucleotide sequences of 13 PCGs, with *Lanius tigrinus* as the outgroup. Support values are denoted next to nodes after 1000 bootstrap replicates.

## Data Availability

The data that support the findings of this study are openly available in the NCBI Genbank database at https://www.ncbi.nlm.nih.gov/genbank/, reference number MT366880.
